# Dietary Fiber Supplementation in Gestating Sow Diet Improved Fetal Growth and Placental Development and Function Through Serotonin Signaling Pathway

**DOI:** 10.3389/fvets.2022.831703

**Published:** 2022-05-12

**Authors:** Yang Li, Min Yang, Lijia Zhang, Zhengyu Mao, Yan Lin, Shengyu Xu, Zhengfeng Fang, Lianqiang Che, Bin Feng, Jian Li, Yong Zhuo, De Wu

**Affiliations:** ^1^Key Laboratory for Animal Disease-Resistance Nutrition of the Ministry of Agriculture, Animal Nutrition Institute, Sichuan Agricultural University, Chengdu, China; ^2^Shandong Provincial Key Laboratory of Animal Biotechnology and Disease Control and Prevention, Department of Animal Science, Shandong Agricultural University, Taian, China; ^3^Pet Nutrition and Health Research Center, Chengdu Agricultural College, Chengdu, China

**Keywords:** gestation, fiber, 5-HT, placenta, reproductive performance, gut microbiota

## Abstract

The experiment was conducted to investigate the effects of dietary fiber (DF) supplementation in gestation diet on fetal growth and placental development and function and explore the possible mechanism of DF improving sow reproductive performance. A total of 16 Large White × Landrace crossbred gilts were randomly allotted to two groups and fed a semi-purified basal diet [non-fiber (NF) group, 0.1% total DF] or a basal diet supplemented with 8.33 g/kg inulin and 200 g/kg cellulose [Fiber (F) group] during the gestation period. On day 106 of gestation, five sows per group were chosen and slaughtered for sample collection. Results showed that DF supplementation during gestation increased the total fetal weight and placental weight on day 106 of gestation; elevated serum serotonin concentration; increased concentrations of serotonin and short-chain fatty acids (acetate, propionate, and butyrate), as well as tryptophan hydroxylase 1 expression, in colon; elevated serotonin and progesterone concentrations and up-regulated the serotonin transporter, cytochrome P450 11A1, and insulin-like growth factor 2 expressions in the placenta. Besides, the sows in the F group had microbial community structures distinct from those in the NF group. Supplementation of DF in gestation diet increased the *Coprococcus 3* abundance that was positively correlated with colonic serotonin concentration, while significantly decreasing the *Family XIII AD3011 group* abundance which was negatively correlated with colonic serotonin concentration. Above all, DF supplementation in the gestation diet could increase placental serotonin levels by promoting maternal serotonin synthesis in the colon and the transport from the mother to the placenta in sows, and then improve placental development and function, finally promoting fetal growth. Our findings provided insight into the mechanisms of DF improving sow reproductive performance.

## Introduction

Many studies have proven that dietary fiber (DF) supplementation during gestation improved sow reproductive performance, such as improved embryo survival, litter size and weight, and uniformity of piglets within litter (1–7). However, most studies have been limited to the effects of DF addition on sow reproduction performance, and only a few studies have explored the mechanism by which DF supplementation improves sow fertility. The placenta is an important organ for material exchange between the fetus and mother. Placenta insufficiency is a common cause that leads to low litter size, birth weight, and uniformity of piglets within litter. A recent study showed that DF supplementation during gestation for a successive three parities could improve litter size and weight and increase placental weight in the second and third parities (2), which suggested that DF supplementation during gestation might improve sow reproductive performance through improving placental development and function. However, it is still unclear how the DF affects placental development and function in sows.

Serotonin, as known as 5-hydroxytryptamine (5-HT), is mainly produced in the gut by enterochromaffin cells (ECs) and is a key monoamine neurotransmitter participating in the modulation of central neurotransmission and enteric physiological function (8). Tryptophan is the sole precursor of serotonin. In the gut, tryptophan is first converted to 5-hydroxytroptophan (5-HTP) by the rate-limiting enzyme, tryptophan hydroxylase 1 (TPH1), and aromatic amino acid decarboxylase (AAAD) subsequently converts 5-HTP into serotonin (9). Gut-derived serotonin can be transported through a serotonin transporter (SERT) to different parts of the body (including the placenta) to regulate diverse functions (10). Many research studies showed that the lack of maternal serotonin could decrease the placental serotonin concentration and lead to placental insufficiency, resulting in inhibition of fetal growth and development (11–13). Zhuo et al. (14) demonstrated that DF supplementation could promote colonic serotonin synthesis and increase the plasma serotonin concentration in sows. Therefore, we speculated that DF supplementation might promote placental development and function through the maternal–placental serotonin signaling pathway, and then improve fetal growth and development in gestating sows.

The objective of the present study was to investigate the effects of DF supplementation in gestation diet on fetal growth, placental development and function, and serotonin signaling pathway, and explore the possible mechanism of dietary fiber improving the reproductive performance of sows.

## Materials and Methods

### Animals, Diets, and Management

A total of 16 Large White × Landrace crossbred gilts with an initial body weight (BW) of 204.88 ± 3.97 kg were bred to Duroc boars on the third estrus and randomly allotted to two dietary treatment groups (*n* = 8 gilts/group). The experimental diets were a semi-purified basal diet [non-fiber diet (NF), Table 1] and a fiber diet (F), which was the basal diet supplemented with 8.33 g/kg inulin (ZTH Tech, Beijing, China) and 200 g/kg cellulose (Guangxi Shangda Tech Co., Nanning, China). The basal diet was formulated to meet or exceed the nutrient requirements of gestating sows by the National Research Council (15). Gestating sows were housed individually in gestation stalls (2.37 m length × 0.65 m width × 1.13 m height). All sows were allowed *ad libitum* access to water and were fed two times each day (0900 and 1600) during gestation. The daily feed amount per sow in the NF group was 2.40 kg/day from 1 to 105 day, and the corresponding daily feed amount per sow in the F group was 2.90 kg/day.

**Table 1:** Composition and calculated analysis of semi-purified basal diet.

**Items**	**Basal diet**
**Ingredient, %**	
Wheat flour	47.89
Corn starch	40.07
Soy protein isolate	7.72
Fish meal, 53.5% CP	1.00
Limestone	0.90
Monocalcium phosphate	1.30
Sodium chloride	0.40
L-lysine HCl, 76.8%	0.02
DL-methionine, 99%	0.10
L-threonine, 98%	0.04
L-tryptophan, 98%	0.01
Choline	0.15
Premix of vitamins and minerals^a^	0.40
Total	100
**Nutrient analysis**	
Digestible energy, MJ/kg	15.48
Crude protein, %	14.08
Crude fat, %	0.42
Starch, %	0.14
Crude fiber, %	0.01
Total dietary fiber, %	0.01
Total calcium, %	0.73
Standardized total tract digestible phosphorus, %	0.34
Standardized ileal digestible lysine, %	0.58
Standardized ileal digestible methionine, %	0.27
Standardized ileal digestible threonine, %	0.39
Standardized ileal digestible tryptophan, %	0.13

a *Provided per Kilogram of Complete Basal Diet: Vitamin A 7500 IU, Vitamin D_3_ 5000 IU, Vitamin E 37.5 IU, Vitamin K_3_ 5 mg, Vitamin B_1_ 5 mg, Vitamin B_3_ 12.5 mg, Vitamin B_6_ 7.5 mg, Vitamin B_12_ 0.05 mg, Biotin 0.2 mg, Niacin 50 mg, Folic Acid 2.5 mg, D-Calcium Pantothenate 25 mg, Cu 10 mg, Fe 100 mg, I 0.6 mg, Zn 100 mg, Mn 30 mg, Se 0.25 mg*.

### Sampling

On day 106 of gestation, fasting blood samples (10 ml) from 10 healthy sows (5 sows per group with similar BW to the average group weight) were collected from the ear vein in the morning, and collected into two tubes (5 ml) without heparin sodium and kept at room temperature for 30 min followed by centrifugation for 15 min at 3,500 × g at 4°C. Serum samples were harvested and stored at −20°C until analysis.

After blood sample collection, the 10 sows were weighed and anesthetized by intramuscularly injecting Zoletil 50 vet at the dose of 0.1 mg/kg BW (Virbac, Carros, France), then the uterus was removed from the sows. The number and weight of fetuses were recorded, and the total fetal weight was calculated as the number of fetuses multiplied by the fetal weight. Subsequently, the placenta was separated manually and weighed. Placenta tissues (2 cm × 2 cm) ~5 cm far away from the joint site of the umbilical cord to the placenta were collected from the male fetus near the average fetal weight each sow. One part of the placenta tissues was snap-frozen in liquid nitrogen immediately, and then stored at −80°C until further analysis; another part was fixed in 4% paraformaldehyde solution for protein expression measurement using immunohistochemistry (IHC) method with specific antibodies.

In addition, the colonic digesta samples were collected on a clean bench, and placed in sterile bags immediately, followed by being stored at −80°C for short-chain fatty acids (SCFAs) analysis. Subsequently, the clean colonic tissues were collected after being washed in ice-cold physiological saline solution and stored at −80°C immediately after being snap-frozen in liquid nitrogen.

### Serotonin Concentration Analysis in the Serum, Colon, and Placenta

Serotonin levels in serum were detected by serotonin ELISA for the quantitative determination of serotonin in serum, plasma, and urine (DLD Diagnostika GmBH, Hamburg, German) according to the manufacturer's instruction, and those in the colon and placenta were detected by Serotonin High Sensitive ELISA for high sensitivity and small sample volume (DLD Diagnostika GmBH) according to the manufacturer's instruction as described previously (14).

### Placental Progesterone Concentration Analysis

Placental progesterone concentration was analyzed using a commercial radioimmunoassay kit purchased from North Institute of Biotechnology Co., Ltd (Beijing, China). The detection steps are as follows. Briefly, the placenta tissues were weighed and homogenized in phosphate buffer solution (pH = 0.72). Then ethane was added to the placenta homogenate and allowed to stand for 45 min at 4°C, followed by supernatant collection. Finally, 10 μl of supernatant was used for the assay of progesterone concentration in the placenta.

### Activities of Tryptophan Hydroxylase and Indoleamine-2, 3-Dioxygenase Concentration Analysis

The activities of TPH and indoleamine-2, 3-dioxygenase (IDO) in the colon were determined using specific assay kits purchased from Nanjing Jiancheng Bioengineering Institute (Nanjing, China) according to the manufacturer's instructions. In brief, 50 μl of the prepared supernatant of tissue homogenate and diluted standard solutions were added to the corresponding microplates. After reacting for 30 min at 37°C, the microplates were washed five times and 50 μl of the HRP–Conjugated Reagent was added to each well. Then the microplates were cultured for 30 min at 37°C and washed five times again. The chromogenic procedure was performed with two kinds of chromogenic agents for 10 min at 37°C followed by Stop Buffer addition. Finally, the absorbance of each well was read within 15 min, and the concentrations of TPH and IDO were calculated using the standard curve made with standard solutions.

### Short-Chain Fatty Acids Analysis in the Colonic Contents

The SCFA concentrations in the colonic contents were measured using a gas chromatographic method as described previously (16). Briefly, fecal samples (about 0.7 g) were suspended in 1.5 ml of distilled water and was allowed to stand for 30 min, followed by being centrifuged at 15,000 × g for 15 min. The supernatant (1 ml) was transferred and mixed with 0.2 ml metaphosphoric acid (25%, w/v) and 23.3 μl crotonic acid (210 mmol/L, internal standard). After standing at 4°C for 30 min, the samples were centrifuged at 15,000 × g for 10 min again. The 0.3 ml supernatant was transferred and mixed with 0.3 ml methanol. A small aliquot of the supernatant (1 μl) was analyzed using gas chromatography (Varian CP-3800 GC, United States).

### Gene Expression

The gene mRNA expression levels of SERT and TPH1 in colonic samples, and the gene mRNA expression levels of SERT, TPH1, scavenger receptor BI (SRBI), low-density lipoprotein receptor (LDLR), cytochrome P450 11A1 (CYP11A1), 3β-hydroxysteroid dehydrogenase/isomerase (3β-HSD), insulin-like growth factor 2 (IGF-2), H19, cysteine-rich 61 (CYR61), and vascular endothelial growth factor (VEGF) in the placental samples were detected using the CFX-96 real-time PCR detection system (Bio-Rad, Hercules, CA, United States). Primer sequences used for real-time PCR were described in Supplementary Table S1. β-Actin was amplified in tandem with the target gene as an internal control, allowing for gene normalization and measurement. The process of measurement was described in a previous study (17). Relative mRNA abundances of the detected genes in the placenta samples were calculated using the 2^−ΔΔ^ Ct method.

### Protein Expression

Colonic and placental TPH1 and SERT protein expressions were measured using the IHC method. Fixed colonic and placental tissues were embedded according to routine paraffin-embedding protocol, followed by being sectioned for 4 μm using a microtome. Prepared sections were incubated with primary antibody for rabbit anti-TPH1 or anti-SERT (Affinity Biosciences, Zhenjiang, Jiangsu, China) after microwave antigen retrieval, endogenous peroxidase blocked and blocked with 3% bull serum albumin (BSA; Solarbio Bioscience &Technology Co., Ltd., Shanghai, China) for 30 min. Then the sections were incubated with horseradish peroxidase (HRP)-labeled secondary antibody for goat anti-mouse immunoglobulin (DAKO, Glostrup, Denmark) after the phosphate buffer solution (PBS) washing and reacted at room temperature for 50 min. The newly prepared 3-3'-diamino-benzidine (DAB) chromogenic reagent (DAKO, Glostrup, Denmark) was added after the PBS washing and the developing time was chosen using a microscope (magnification, 400 ×, Nikon Eclipse Ti-SR, Japan). The reaction was terminated with tap water. After being counterstained with Harris hematoxylin, differentiated with 1% hydrochloric acid alcohol and showed blue with ammonia, the samples were dehydrated with alcohol and cleared in xylene, and the slices were sealed with neutral gum. Three sections per specimen and 5 random fields per section were analyzed by 2 independent observers using ImageJ software to measure the optical density for quantifying the protein expression of TPH1 and SERT in the colon and placenta.

### 16S RRNA Gene Sequencing

Bacterial genomic DNA was extracted from frozen sow fecal samples with an E.Z.N.A. TM Stool DNA kit (Omega Bio-Tek, Norcross, GA, United States). Sequencing and data analysis were subsequently performed on the Illumina HiSeq PE250 platform by Novogene Institute (Beijing, China), as previously described in Li et al. (18). The V3–V4 region of 16S rDNA was amplified with the barcoded V4: 515F-806R primers (5′-GTGCCAGCMGCCGCGGTAA-3′ and 5′-GGACTACHVGGGTWTCTAAT-3′, respectively). All sequencing data are available in the NCBI Sequence Read Archive (SRA) under accession PRJNA_783061 (Illumina sequences). Sequences were clustered into the same operational taxonomic units (OTUs) with a 97% similarity threshold. Chao 1 index, Shannon index, and Simpson index were used to ascertain differences in the alpha diversity, and Wilcoxon rank-sum test was used to detect the statistical differences between the two groups. Bray–Curtis distance matrices were calculated for comparison of taxonomic data in beta diversity, and analysis of similarities (ANOSIM) was used to access differences among the microbial communities. All analyses from clustering to alpha and beta diversity were performed in QIIME (V1.7.0) and displayed in R software (V2.15.3).

### Statistical Analysis

The sow was considered as the experimental unit for all variables, and the *t*-test procedure of SAS 9.4 (Institute Inc., Cary, NC, United States) was used to evaluate the significance between the two groups after assessment of normality of the data using Shapiro–Wilk's statistic (*W* > 0.05). The total number of piglets was included as a covariate in the analysis of fetal and placental weight. Spearman's correlations were used to assess the associations between bacterial abundance and colonic serotonin concentration. Values are expressed as mean ± standard error (SE) in tables and figures. Statistical significance was set at *P* < 0.05, and 0.05 < *P* < 0.10 was considered a trend toward significance.

## Results

### Effects of Fiber Supplementation on Fetus Number, Fetal Weight, and Placental Weight

**Figure 1:**
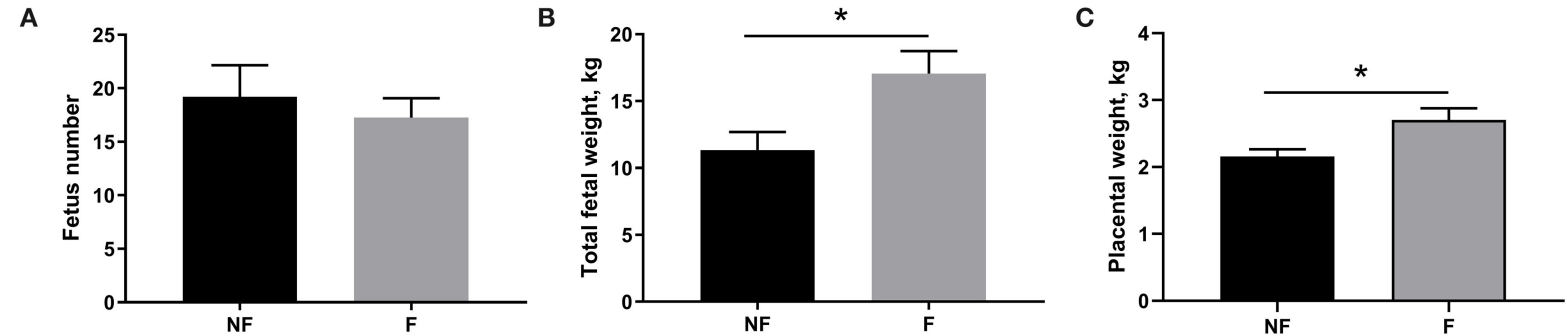
Effects of fiber supplementation in gestating sow diet on fetus number, fetal weight, and placental weight. **(A)** Fetus number; **(B)** Total fetal weight; **(C)** Placental weight. NF, sows fed with a semi-purified basal diet (0.01% dietary fiber); F, sows fed with a semi-purified basal diet supplemented with 8.33 g/kg inulin and 200 g/kg cellulose. Values are mean ± standard error (*n* = 5). Significant differences are displayed in the figures by *P < 0.05.

Effects of fiber supplementation on sow reproductive performance are presented in Figure 1. Dietary fiber supplementation during gestation significantly increased the total fetal weight (*P* < 0.05) and placental weight (*P* < 0.05) on day 106 of gestation, but had no significant effect on fetus number (*P* > 0.05).

### Effects of Fiber Supplementation on Maternal and Placental Serotonin Concentration

**Figure 2:**
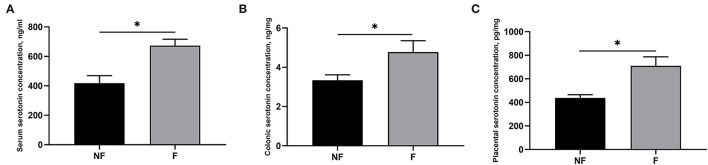
Effects of fiber supplementation in gestation diet on serotonin concentrations in serum, colon, and placenta of the sow. **(A)** Serum serotonin; **(B)** Colonic serotonin; **(C)** Placental serotonin. NF, sows fed with a semi-purified basal diet (0.01% dietary fiber); F, sows fed with a semi-purified basal diet supplemented with 8.33 g/kg inulin and 200 g/kg cellulose. Values are mean ± standard error (*n* = 5). Significant differences are displayed in the figures by *P < 0.05.

Effects of fiber supplementation on the serotonin concentration in serum, colon, and placenta are shown in Figure 2. Sows fed the diet supplemented with DF had significantly higher serotonin concentration in serum, colon, and placenta compared with those fed the diet without fiber on day 106 of gestation (*P* < 0.05).

### Effects of Fiber Supplementation on Serotonin Synthesis and Transport in Sow Colon

**Figure 3:**
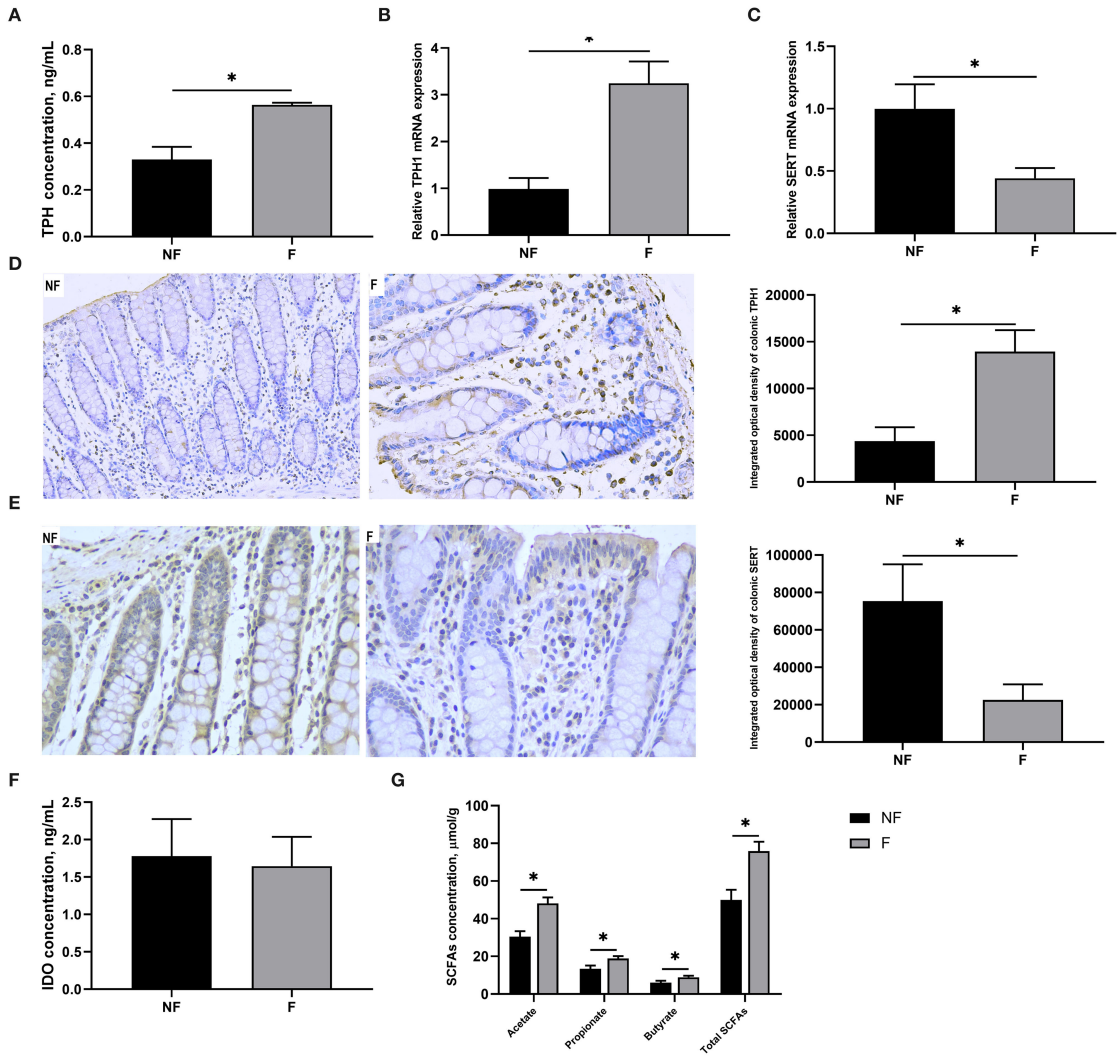
Effects of fiber supplementation in gestation diet on serotonin synthesis and transport in sow colon. **(A)** TPH concentration; **(B,C)** Gene expression of TPH1 and SERT; **(D,E)** Protein expression of TPH1 and SERT measured using immunohistochemistry; **(F)** IDO concentration; **(G)** SCFAs concentration. NF, sows fed with a semi-purified basal diet (0.01% dietary fiber); F, sows fed with a semi-purified basal diet supplemented with 8.33 g/kg inulin and 200 g/kg cellulose. TPH, tryptophan hydroxylase; SERT, serotonin transporter; IDO, indoleamine-2, 3-dioxygenase; SCFAs, short-chain fatty acids. Values are mean ± standard error (*n* = 5). Significant differences are displayed in the figures by * *P* < 0.05.

As shown in Figure 3, DF supplementation during gestation significantly increased colonic concentration of TPH (*P* < 0.05) and mRNA expression of TPH1 (*P* < 0.05), but significantly decreased mRNA expression of SERT (*P* < 0.05). Consistently, IHC results also showed that sows fed the diet supplemented with DF had higher protein expression of TPH1 (*P* < 0.05) but lower protein expression of SERT (*P* < 0.05) in the colon than those fed the diet without DF (*P* < 0.05). Besides, the colonic concentrations of acetate, propionate, butyrate, and total SCFAs in the F sows were all significantly higher than in the NF sows (*P* < 0.05). However, no significant difference was observed in the colonic IDO concentration between the two groups (*P* > 0.05).

### Effects of Fiber Supplementation on Serotonin Synthesis and Transport in Sow Placenta

**Figure 4:**
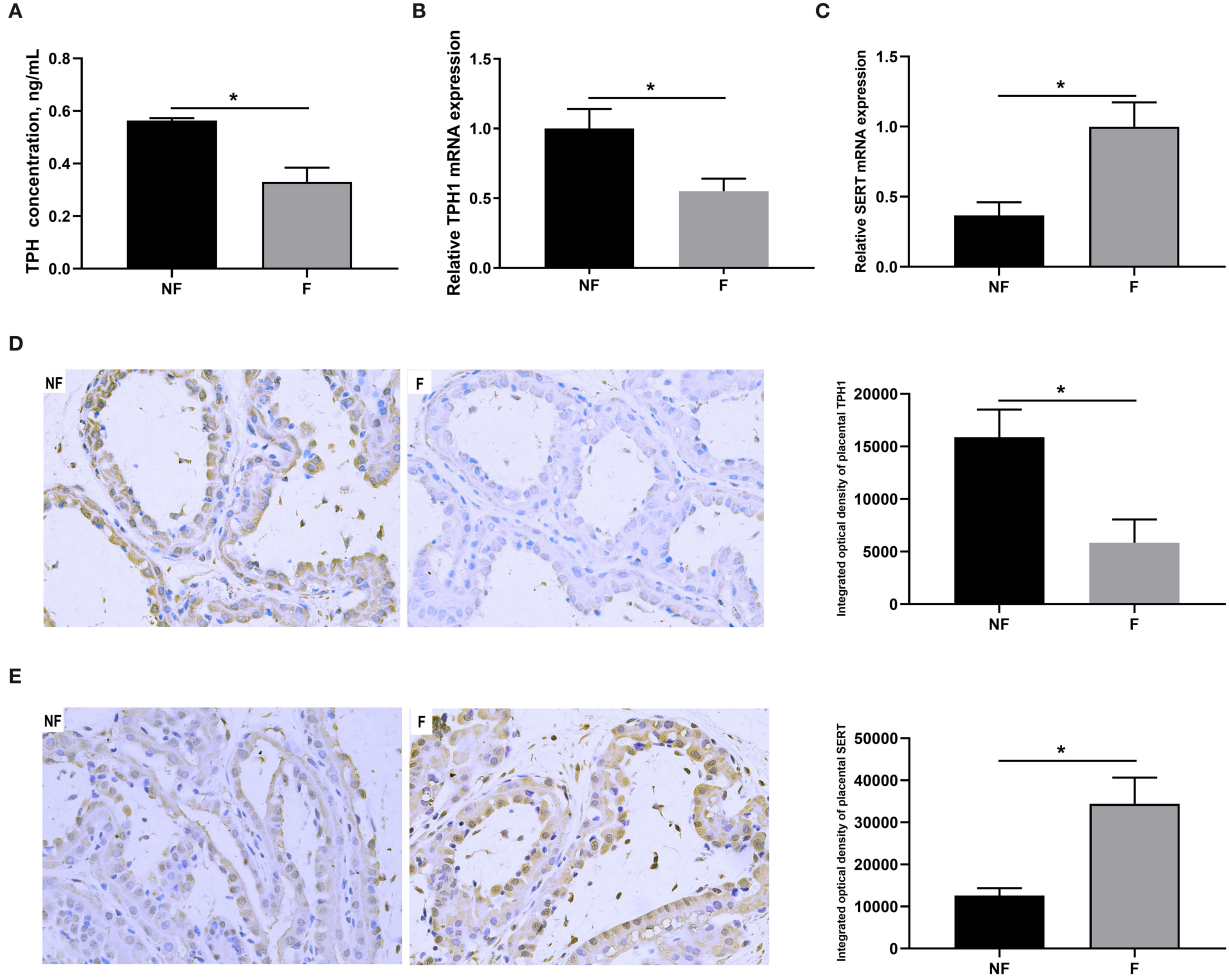
Effects of fiber supplementation in gestation diet on serotonin synthesis and transport in sow placenta. **(A)** TPH concentration; **(B,C)** Gene expression of TPH1 and SERT; **(D,E)** Protein expression of TPH1 and SERT measured using immunohistochemistry. NF, sows fed with a semi-purified basal diet (0.01% dietary fiber); F, sows fed with a semi-purified basal diet supplemented with 8.33 g/kg inulin and 200 g/kg cellulose. TPH, tryptophan hydroxylase; SERT, serotonin transporter. Values are mean ± standard error (*n* = 5). Significant differences are displayed in the figures by * *P* < 0.05.

As shown in Figure 4, DF supplementation during gestation significantly decreased placental concentration of TPH (*P* < 0.05) and mRNA expression of TPH1 (*P* < 0.05), but significantly increased mRNA expression of SERT (*P* < 0.05). Consistently, IHC results also showed that sows fed the diet supplemented with DF had lower protein expression of TPH1 (*P* < 0.05) but higher protein expression of SERT (*P* < 0.05) in the placenta than those fed the diet without DF (*P* < 0.05).

### Effects of Fiber Supplementation on Progesterone Synthesis in Sow Placenta

**Figure 5:**
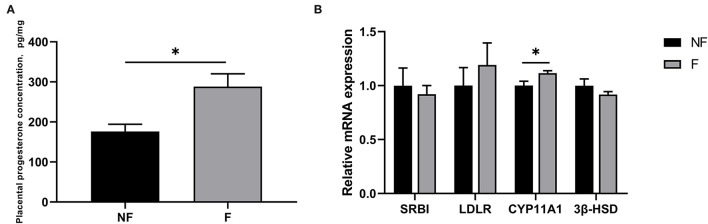
Effects of fiber supplementation in gestation diet on progesterone concentration and gene expressions related to progesterone synthesis in sow placenta. **(A)** Progesterone concentration; **(B)** Gene expressions of scavenger receptor BI (SRBI), low-density lipoprotein receptor (LDLR), cytochrome P450 11A1 (CYP11A1), and 3β-hydroxysteroid dehydrogenase/isomerase (3β-HSD). NF, sows fed with a semi-purified basal diet (0.01% dietary fiber); F, sows fed with a semi-purified basal diet supplemented with 8.33 g/kg inulin and 200 g/kg cellulose. Values are mean ± standard error (*n* = 5). Significant differences are displayed in the figures by * *P* < 0.05.

The progesterone concentration and the gene expressions related to progesterone synthesis in the placenta are shown in Figure 5. Dietary fiber supplementation during gestation significantly increased the progesterone concentration and the mRNA expression of CYP11A1 in the placenta (*P* < 0.05). There were no significant differences in the mRNA expressions of SRBI, LDLR, and 3β-HSD (*P* > 0.05).

### Effects of Fiber Supplementation on the Expressions of Genes Related to Sow Placental Development

**Figure 6:**
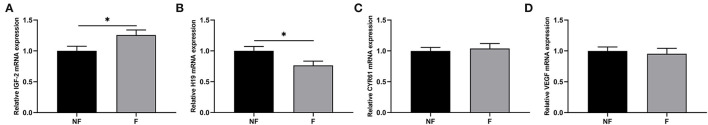
Effects of fiber supplementation in gestation diet on gene expressions related to placental development of sow. **(A)** Insulin-like growth factor 2 (IGF-2); **(B)** H19; **(C)** Cysteine-rich 61 (CYR61); **(D)** Vascular endothelial growth factor (VEGF). NF, sows fed with a semi-purified basal diet (0.01% dietary fiber); F, sows fed with a semi-purified basal diet supplemented with 8.33 g/kg inulin and 200 g/kg cellulose. Values are mean ± standard error (*n* = 5). Significant differences are displayed in the figures by * *P* < 0.05.

Effects of fiber supplementation on the expressions of genes related to placental development are shown in Figure 6. Dietary fiber supplementation significantly increased the IGF-2 mRNA expression (*P* < 0.05) but significantly decreased the H19 mRNA expression in the placenta (*P* < 0.05). There were no significant differences in the mRNA expressions of CYR61 and VEGF (*P* > 0.05).

### Effects of Fiber Supplementation on Microbial Diversity in Colonic Digesta

**Figure 7:**
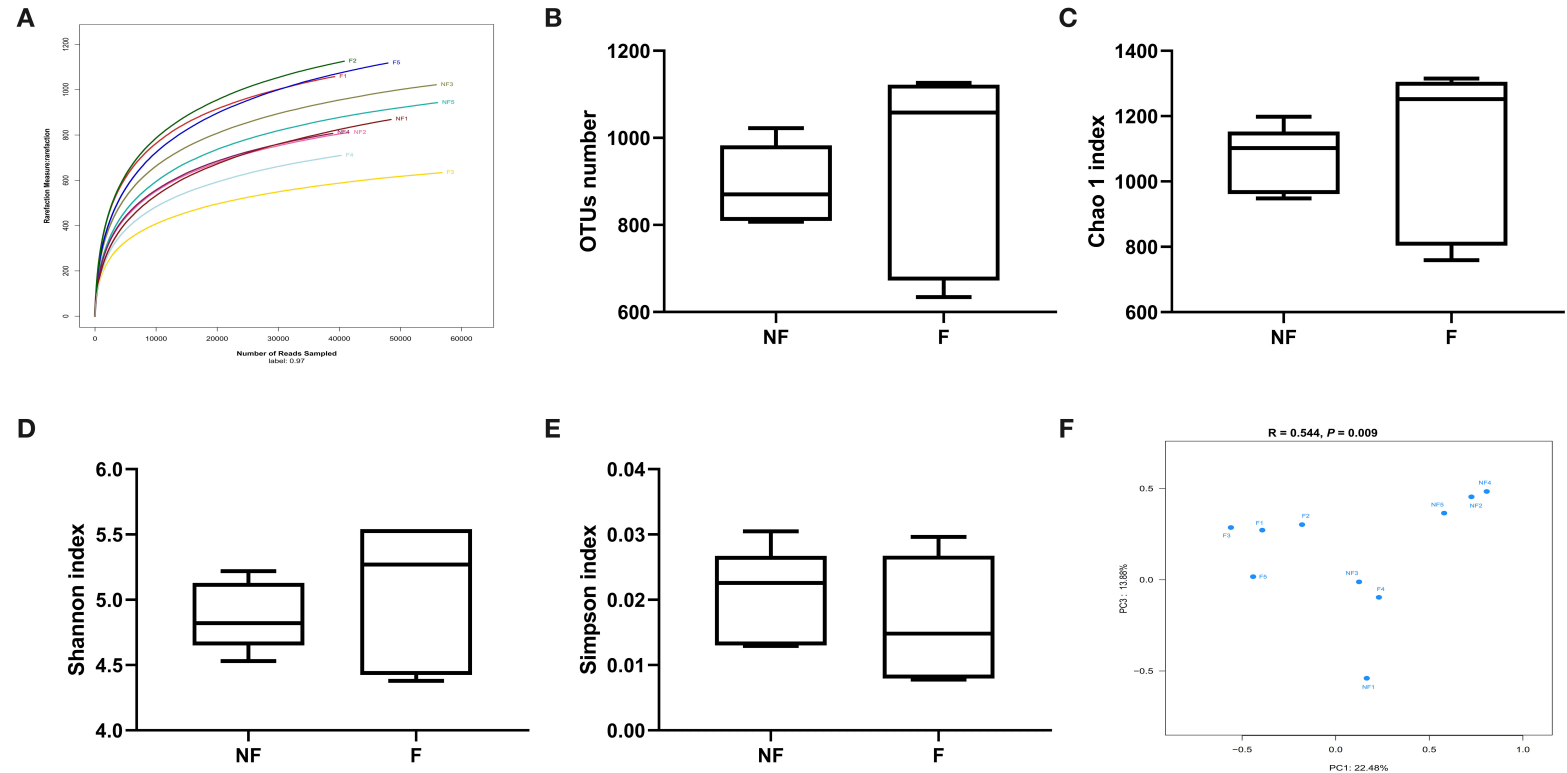
Effects of fiber supplementation in gestation diet on bacteria community diversity and richness between the two groups. **(A)** Rarefaction curve; **(B)** Number of operational taxonomic units (OTUs); **(C)** Chao 1 index; **(D)** Shannon index; **(E)** Simpson index; **(F)** The principal coordinate analysis (PCoA) profile of the two groups displayed with Bray_curtis distance, and analysis of similarities (ANOSIM) was used to evaluate differences among the microbial communities. NF, sows fed with a semi-purified basal diet (0.01% dietary fiber); F, sows fed with a semi-purified basal diet supplemented with 8.33 g/kg inulin and 200 g/kg cellulose. NF 1, 2, 3, 4, and 5 means colonic digesta samples from sows fed NF diet; F 1, 2, 3, 4, and 5 means colonic digesta samples from sows fed F diet. *n* = 5 per group. Significant differences are displayed in the figures by * *P* < 0.05.

The rarefaction curve (Figure 7A) was used to estimate the species richness of each sample. The OTU number tended to be smooth with the increasing number of the sequence, indicating that the sequencing depth was enough for OTUs testing. No significant differences were observed in OUT number (Figure 7B), Chao 1 index (Figure 7C), Shannon index (Figure 7D), and Simpson index (Figure 7E) between the two treatments (*P* > 0.05). However, the PCoA profile based on Bray–Curtis distance (Figure 7F) showed a clear separation between the NF and F groups, and the ANOSIM analysis also demonstrated that the two groups had significantly different microbiota community structures (*R* = 0.544, *P* = 0.009).

### Effects of Fiber Supplementation on Microbial Relative Abundance in Colonic Digesta

**Figure 8:**
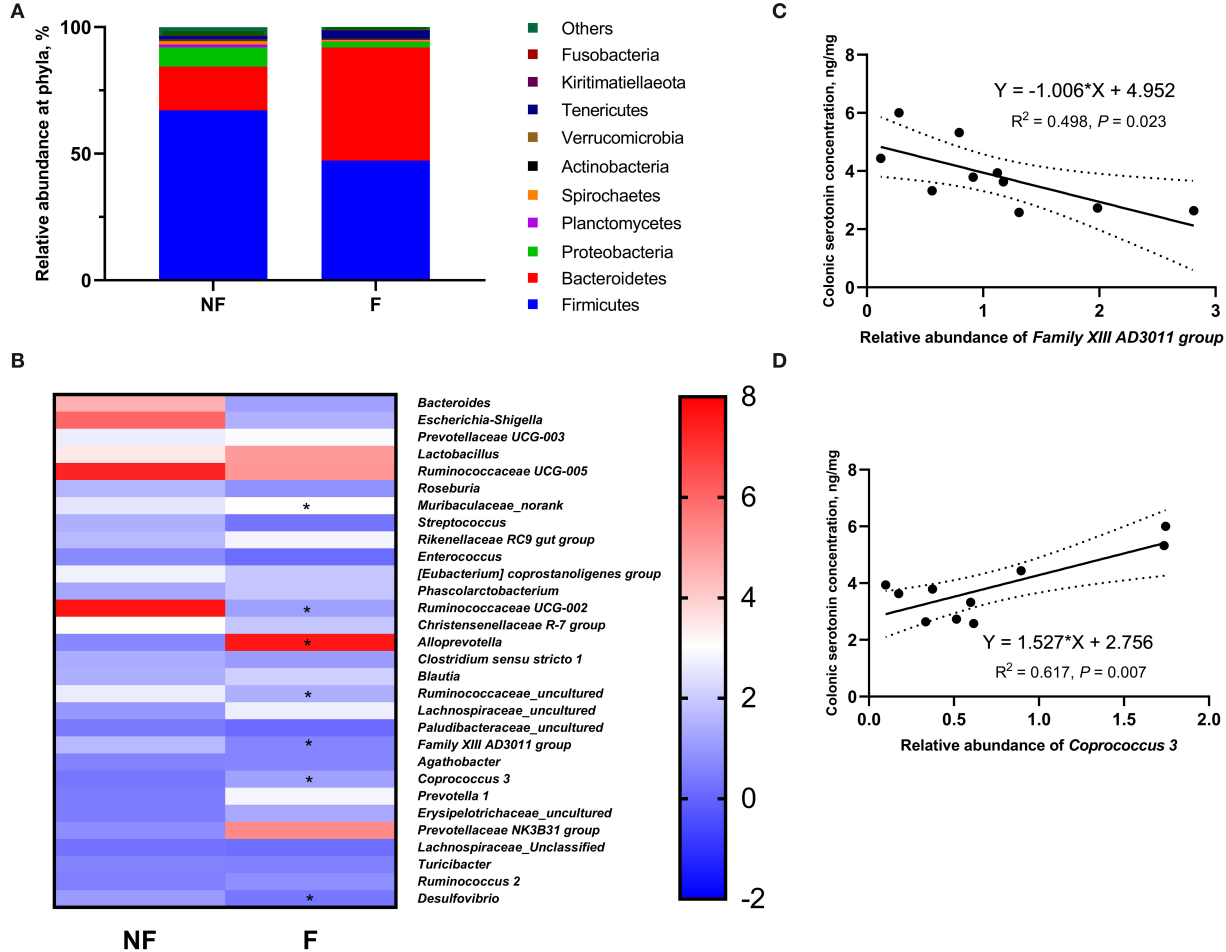
Effects of fiber supplementation in gestation diet on relative abundances of colonic microbiota in sows. **(A)** Relative abundance of colonic microbiota at phylum level; **(B)** Relative abundance of colonic microbiota at genus level; **(C,D)** Correlation analysis between the colonic serotonin concentration and the colonic microbiota that showed significant differences between the two groups. NF, sows fed with a semi-purified basal diet (0.01% dietary fiber); F, sows fed with a semi-purified basal diet supplemented with 8.33 g/kg inulin and 200 g/kg cellulose. *n* = 5 per group. Significant differences are displayed in the figures by * *P* < 0.05.

The relative abundances of top ten phyla in sow colonic digesta are shown in Supplementary Table S2 and Figure 8A. The most predominant phyla were Firmicutes and Bacteroidetes. Sows fed the F diet had a significantly lower relative abundance of Firmicutes (*P* < 0.05), but a higher relative abundance of Bacteroidetes (*P* < 0.05) than sows fed the NF diet. Besides, DF supplementation during gestation tended to decrease the Proteobacteria abundance compared with that of the NF group (*P* < 0.10).

The relative abundance at the genus level in sow colonic digesta (top 35) is shown in Supplementary Table S3 and Figure 8B. Dietary fiber supplementation significantly increased the relative abundances of *Muribaculaceae_norank, Alloprevotella*, and *Coprococcus 3* (*P* < 0.05) but significantly decreased the relative abundances of *Ruminococcaceae UCG-002, Ruminococcaceae_uncultured, Family XIII AD3011 group*, and *Desulfovibrio* (*P* < 0.05). Moreover, the abundance of *Christensenellaceae R-7 group* in the F group tended to be lower than that in the NF group (*P* < 0.10).

### Correlation Analysis Between Fecal Microbial Abundance and Colonic Serotonin Concentration

Of the seven genera that showed significant differences between the two groups, the relative abundance of *Family XIII AD3011 group* had a significantly negative correlation with colonic serotonin concentration (*P* < 0.05, Figure 8C); the relative abundance of *Coprococcus 3* had a significantly positive correlation with colonic serotonin concentration (*P* < 0.05, Figure 8D).

## Discussion

In the present study, we found that DF supplementation during gestation increased the total fetal weight on day 106 of gestation when the daily nutrient intake except DF was equal to the NF group. Similarly, previous studies also showed that sows fed a high-fiber diet during gestation had a higher litter weight than sows fed a low-fiber diet over two or three successive reproductive cycles (2, 19, 20). However, the mechanism that addition of DF in gestating sow diet improves fetal development remains unclear. The placenta, as the interface between mother and fetus, is central to prenatal growth control. Placenta insufficiency will reduce nutrient and oxygen delivery and cause predictably poor intrauterine conditions for the fetus (21). Recently, Li et al. (2) demonstrated that fiber supplementation increased the placental weight, which might be contributed to the increased litter weight. Consistently, we also found increased placental weight in the sows fed the F diet compared with the sows fed the NF diet in the present study. Moreover, DF supplementation in the gestation diet increased sow placental IGF-2 mRNA expression and decreased H19 mRNA expression. Insulin-like growth factor 2 and H19 are a couple of imprinting genes that are involved in fetal and placental development (22). H19 is a parenterally imprinted maternally expressed gene, which is not translated to protein and functions as an RNA molecule, and is closely related to the oppositely imprinted expressed IGF-2 (23). A previous study in the mice showed that deleting placental-specific IGF-2 resulted in decreased fetal and placental weights, as well as a reduced surface area of the exchange barrier in the labyrinth of the mouse placenta (24), suggesting that up-regulated IGF-2 contributed to the development of fetus and placenta. Moreover, mice lacking H19 showed an overgrowth phenotype, indicating that H19 could affect fetal development by inhibiting excessive growth of the placenta (25, 26). The results of IGF-2 and H19 mRNA expressions demonstrated that fiber supplementation in the gestation diet could promote sow placental development by regulating the gene expression of imprinted genes IGF-2 and H19.

Progesterone is a steroid hormone crucial for placental development and fetal growth. A previous study *in vitro* demonstrated that progesterone could stimulate IGF-2 production in human osteoblastic cells (27). The placenta is the primary site of progesterone synthesis and secretion in late pregnancy. In the placental inner mitochondrial membrane, the maternal cholesterol is first converted into pregnenolone by CYP11A1, which is the rate-limiting step of placental progesterone synthesis, and then pregnenolone is metabolized into progesterone by 3β-HSD (28). In the present study, a higher placental progesterone level was observed in the sows fed the F diet, which might be benefited from increased CYP11A1 expression. Therefore, DF supplementation during gestation could promote the synthesis of placental progesterone by up-regulating the gene expression of CYP11A1.

More recent research studies have shown that the serotonin signaling pathway plays an important role in regulating fetal and placental development (12, 29). The vast majority of serotonin (>90%) in the body of mammals is produced by the ECs (10), where it is synthesized by the TPH1 and stored in secretory granules before release (30). The SERT is responsible for transporting ECs-derived serotonin to different parts of the body to regulate diverse functions (10). In the present study, we found that fiber supplementation in the gestation diet increased the serotonin concentration in the serum and colon, and the activity and expression of colonic TPH1, which might be contributed to the increased concentrations of acetate, propionate, and butyrate. It is well known that DF cannot be digested by mammals but can be fermented by the gut microbiota into SCFAs. Studies demonstrated that gut microbiota-derived SCFAs (including acetate, propionate, and butyrate) promoted the expression of TPH1 in the colon (31, 32). Our current study also demonstrated that fiber supplementation might regulate the synthesis of colonic serotonin by changing colonic microbiota abundance including decreasing *Family XIII AD3011 group* and increasing *Coprococcus 3*. Dias et al. showed that *Family XIII AD3011* often accompanied metabolic disease and inflammation (33). Under the condition of inflammation, IDO activity could be enhanced, and promoted the kynurenine pathway (another physiological pathway for tryptophan metabolism), leading to inhibition of the conversion of tryptophan into serotonin (34). *Coprococcus* is an oxygen-intolerant genus with species that are capable of the SCFAs, which has been shown to promote colon serotonin synthesis (35). In addition, *Coprococcus 3* was reported to have a negative correlation with an inflammatory response (36). Therefore, the gut microbiota was involved in fiber regulating the synthesis of colonic serotonin in the present study. Consistently, Zhuo et al. (14) also found that sows fed the high-fiber diet had significantly higher plasma and colonic serotonin concentrations, as well as colonic TPH1 mRNA expression than sows fed the control diet. Interestingly, we found that sows fed the NF diet displayed elevated colonic expression of SERT than sows fed the F diet, which might be a compensatory response by the body to compensate for the deficient serotonin synthesis. Similarly, Yano et al. (32) found that germ-free mice that had lower serum serotonin concentration exhibited decreased TPH1 expression but increased SERT expression in the colon compared with specific pathogen-free control. Therefore, DF supplementation in the gestation diet could promote the synthesis of colonic serotonin by regulating the production of SCFAs and the composition of gut microbiota.

Koren et al. (37) identified the presence of serotonin in the placenta and showed that placental serotonin played an important role in the maintenance of pregnancy. Studies *in vitro* demonstrated that the addition of serotonin could stimulate progesterone production in cow luteal cells (38) and human granulosa cells (39). Recent research studies showed that the concentration of placental serotonin was reported to be related to fetal and placental development, and the lack of placental serotonin would result in fetal growth restriction (11, 40). The placental serotonin may come from two sources, one of which comes from the mother. Muller et al. (13) showed that inhibition of maternal SERT expression decreased placental 5-HT levels, suggesting that maternal blood 5-HT can freely cross the placental barrier through SERT. Maternal serotonin was reported to play an important role in maintaining normal placental structure and function and promoting fetal development (12, 13, 41). Another source of placental serotonin is synthesized by the placenta itself. Previous studies found that TPH1 and SERT were expressed in the placenta of humans and mice, indicating the placenta itself was capable of producing serotonin using maternal tryptophan (42). However, maternal serotonin plays a major role in regulating fetal and placental development during pregnancy, especially in early pregnancy during which the placenta was not yet capable of synthesizing serotonin (12, 29). For instance, the studies showed suppressed embryonic development in the knockout mice lacking the TPH1 gene (12), and placental dysfunction in regulating metabolic functions and blood coagulation in SERT knockout mouse (29). In the present study, we also found that TPH1 and SERT were expressed in sow placenta using the IHC method. Interestingly, TPH1 and SERT expressions in the placenta of sows fed the fiber diet showed opposite results compared with those in the colon. The elevated placental SERT expression meant that more serotonin could be transported from the mother to the placenta in the F sows, promoting the growth and development of the fetus and placenta. Meanwhile, the increased placental TPH1 in the NF sows might compensate for the decreased serotonin level in the placenta caused by fiber deprivation, which might be also a compensatory response by the body. Therefore, DF supplementation in the gestation diet could increase the placental serotonin concentration by promoting the transport of maternal serotonin to the placenta *via* elevating the SERT expression.

**Figure 9:**
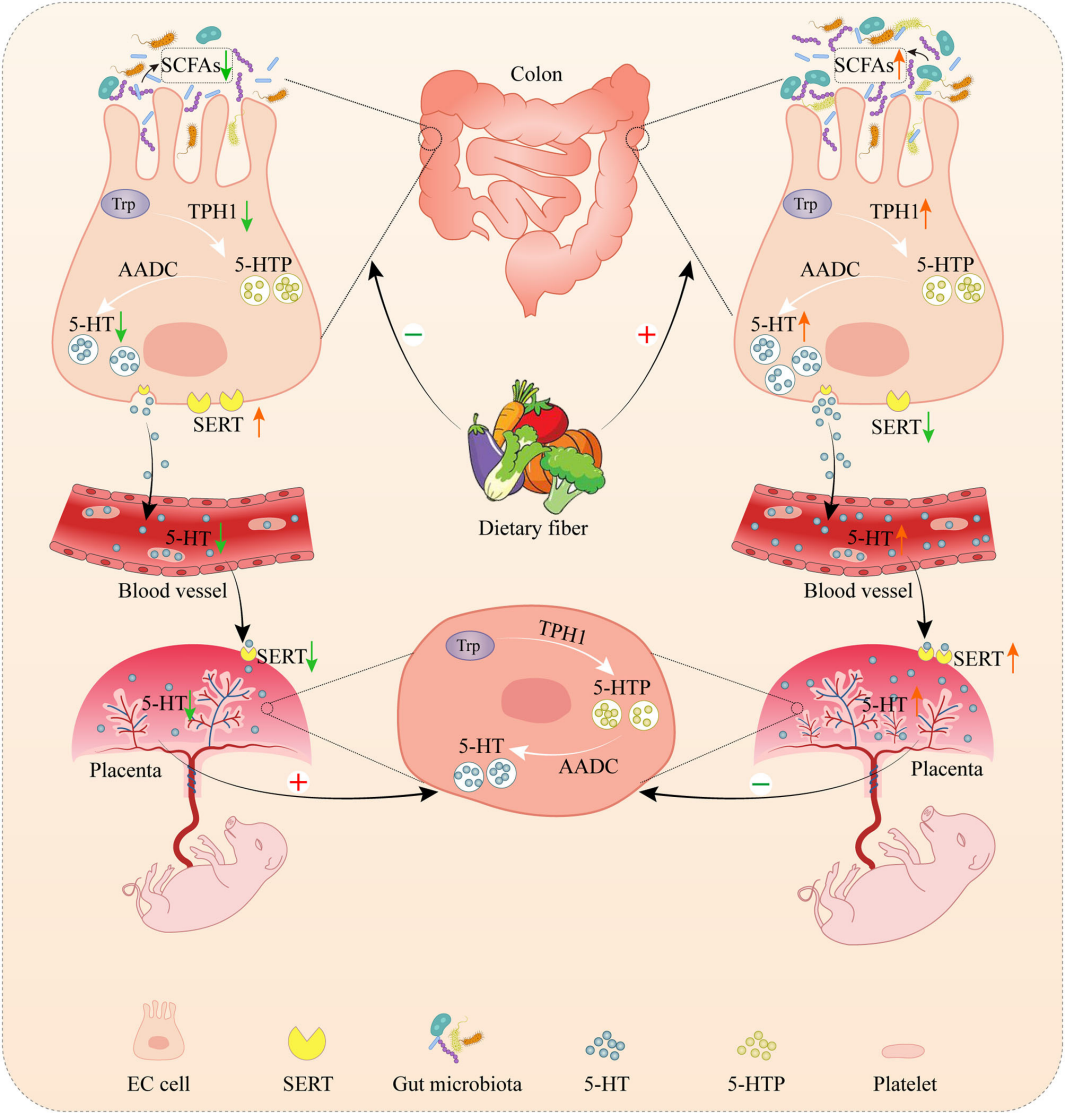
The role of dietary fiber in regulating serotonin signaling pathway and placental and fetal development in gestating sows. Serotonin, as known as 5-hydroxytryptamine (5-HT), is mainly produced in the gut by enterochromaffin cells (ECs). In the gut, tryptophan is first converted to 5-hydroxytroptophan (5-HTP) by tryptophan hydroxylase 1 (TPH1), and aromatic amino acid decarboxylase (AAAD) subsequently converts 5-HTP into serotonin. Gut-derived serotonin can be transported into platelets via serotonin transporter (SERT) which allows the distribution of gut-derived serotonin to peripheral tissues (including the placenta) to regulate diverse functions. The placenta was also capable of producing serotonin. Dietary fiber supplementation increased the colonic concentration by promoting serotonin synthesis in the colon by changing the microbial composition and short-chain fatty acids (SCFAs), leading to an increase in the concentration of maternal serotonin. Meanwhile, dietary fiber supplementation enhanced the serotonin transport from the mother to the placenta, and then increased the placental serotonin concentration, accompanied by the promotion of fetal and placental development. The deprivation of dietary fiber inhibited the serotonin synthesis in the colon and the serotonin transport from the mother to the placenta, leading to the decrease of serotonin in the placenta. However, the deprivation of dietary fiber increased the serotonin transport from the colon to the blood and the serotonin synthesis in the placenta, which might be a compensatory response by the body to compensate for the deficient serotonin in the mother and placenta.

In conclusion, DF supplementation during gestation could increase maternal serum serotonin levels by promoting colonic serotonin synthesis which the gut microbiota might be involved in. Besides, DF supplementation during gestation promoted the serotonin transport from the mother to the placenta in sows, improved the placental development and function, and finally promoted fetal growth (Figure 9).

## Data Availability Statement

The datasets presented in this study can be found in online repositories. The names of the repository/repositories and accession number(s) can be found in the article/Supplementary Material.

## Ethics Statement

The animal study was reviewed and approved by Animal Care and Use Committee of Sichuan Agricultural University.

## Author Contributions

YLi, YZ, and DW: conceptualization. YLi, MY, LZ, ZM, and JL: data curation and project administration. YLi and BF: formal analysis. DW: funding acquisition. YLin and ZF: investigation. MY, LZ, SX, LC, and YZ: methodology. SX, ZF, and YZ: resources. MY, ZM, and YZ: software. YZ and DW: supervision. YLin, LC, and BF: visualization. YLi: writing—original draft. DW: writing—review and editing. All authors have read and agreed to the published version of the manuscript.

## Funding

This research was funded by the National Natural Science Foundation of China (Grant Number 31772616) and the Sichuan Swine Innovation Team of the National Modern Agricultural Industry Technology System (Grant Number scsztd-2021-08-03).

## Conflict of Interest

The authors declare that the research was conducted in the absence of any commercial or financial relationships that could be construed as a potential conflict of interest.

## Publisher's Note

All claims expressed in this article are solely those of the authors and do not necessarily represent those of their affiliated organizations, or those of the publisher, the editors and the reviewers. Any product that may be evaluated in this article, or claim that may be made by its manufacturer, is not guaranteed or endorsed by the publisher.

## Supplementary Material

Supplementary Table S1 The primer sequences of target genes and house-keeping gene.

Supplementary Table S2 Effects of fiber supplementation in gestation diet on microbial relative abundance at phylum level in colonic digesta of sows.

Supplementary Table S3 Effects of fiber supplementation in gestation diet on microbial relative abundance at genus level in colonic digesta of sows.
