# Supplementary material for: Dietary Fiber Supplementation in Gestating Sow Diet Improved Fetal Growth and Placental Development and Function Through Serotonin Signaling Pathway
Source: Front Vet Sci. 2022 May 12;9:831703. doi: 10.3389/fvets.2022.831703 (PMC9133666; doi:10.3389/fvets.2022.831703)
Supplement: Supplementary Table S1 — The primer sequences of target genes and house-keeping gene. [file Table_1.DOCX]

**Table S1.** The primer sequences of target genes and house-keeping gene.

| Genes ^2^ | Primer sequences ^1^ | Size, bp | Gene bank No. |
| --- | --- | --- | --- |
| β-actin | F: AACTGGAACGGTGAAGGTGA | 174 | AY550069.1 |
|  | R: CTTTTGGAAAGGCAGGGACT |  |  |
| TPH1 | F: CCGTCCTGTGGCTGGTTACTTATC | 97 | [XM_021083266.1](https://www.ncbi.nlm.nih.gov/entrez/viewer.fcgi?db=nucleotide&id=1191856929) |
|  | R: GTCCGAACCGTGTCTCACATACTG |  |  |
| SERT | F: CTGTGTGACCTCTCTGGCTC | 129 | [XM_021067520.1](https://www.ncbi.nlm.nih.gov/entrez/viewer.fcgi?db=nucleotide&id=1191822612) |
|  | R: GTGGCGAGCTCATTCATTCT |  |  |
| SRBI | F: TCGCCACACCTCCACAAC | 130 | AF467889 |
|  | R: CCCAAGACCAGAAGCCCG |  |  |
| LDLR | F: GGATAAGCACAGATGCGAAGATA | 177 | NM001206354 |
|  | R: CGGTTGGTGAAGAAGAGGTAGG |  |  |
| 3β-HSD | F: CGTGGATGTGGGTGTGAGG | 85 | AF232699 |
|  | R: TGTATGAAGCCAGTGGCGG |  |  |
| CYP11A1 | F: GCCGCATGGGACACTATTTT | 120 | NM214427.1 |
|  | R: ATTTCCCAGGAGGCGGTAGA |  |  |
| IGF-2 | F: ACACCCTCCAGTTTGTCTGCG | 109 | X56094 |
|  | R: CAGCTACGGAAGCAGCACTCT |  |  |
| H19 | F: TTCCTTGGAGGCTGTTCTGCT | 110 | AY044827 |
|  | R: ACGGTTTCTCATTTTGCCTTTAC |  |  |
| CYR61 | F: GAGCCTCGCGTTCTCTACAC | 310 | [XM_021068763.1](https://www.ncbi.nlm.nih.gov/entrez/viewer.fcgi?db=nucleotide&id=1191825563) |
|  | R: CATCTCTTGCCCTTCTGGAG |  |  |
| VEGF | F: GTCTGGAGTGTGTGCCCA | 104 | XM_013977975.1 |
|  | R: GTGCTGTAGGAAGTCCATC |  |  |

^1^F, forward; R, reverse.

^2^ TPH, tryptophan hydroxylase; SERT, serotonin transporter; SRBI, scavenger receptor BI; LDLR, low-density lipoprotein receptor; CYP11A1, cytochrome P450 11A1; 3β-HSD, 3β-hydroxysteroid dehydrogenase/isomerase; IGF-2, insulin-like growth factor 2; CYR61, cysteine-rich 61; VEGF, vascular endothelial growth factor.

**Table S2.** Effects of fiber supplementation in gestation diet on microbial relative abundance at phylum level in colonic digesta of sows.

| Items, % | Treatment ^1^ | | *P* value |
| --- | --- | --- | --- |
|  | NF | F |  |
| Firmicutes | 67.14 ± 4.62 | 47.25 ± 6.13 | 0.032 |
| Bacteroidetes | 17.21 ± 4.33 | 44.68 ± 6.72 | 0.009 |
| Proteobacteria | 7.63 ± 2.20 | 2.16 ± 0.56 | 0.066 |
| Tenericutes | 1.34 ± 0.84 | 3.29 ± 1.14 | 0.207 |
| Planctomycetes | 1.12 ± 0.52 | 0.14 ± 0.04 | 0.134 |
| Spirochaetes | 1.38 ± 0.53 | 0.70 ± 0.23 | 0.274 |
| Actinobacteria | 0.25 ± 0.07 | 0.27 ± 0.08 | 0.878 |
| Verrucomicrobia | 0.28 ± 0.16 | 0.15 ± 0.10 | 0.515 |
| Kiritimatiellaeota | 0.31 ± 0.15 | 0.22 ± 0.08 | 0.605 |
| Fusobacteria | 0.03 ± 0.01 | 0.03 ± 0.03 | 0.986 |

^1^ NF, sows fed with a semi-purified basal diet (0.01% dietary fiber); F, sows fed with a semi-purified basal diet supplemented with 8.33 g/kg inulin and 200 g/kg cellulose.

Values are mean ± standard error (*n* = 5). Differences between treatments were considered significant at *P* < 0.05.

**Table S3.** Effects of fiber supplementation in gestation diet on microbial relative abundance at genus level in colonic digesta of sows.

| Items, % | Treatment ^1^ | | *P* value |
| --- | --- | --- | --- |
|  | NF | F |  |
| *Bacteroides* | 4.52 ± 2.29 | 1.14 ± 0.60 | 0.190 |
| *Escherichia-Shigella* | 5.99 ± 2.36 | 1.43 ± 0.48 | 0.125 |
| *Prevotellaceae UCG-003* | 2.62 ± 1.47 | 2.93 ± 0.66 | 0.853 |
| *Lactobacillus* | 3.43 ± 1.31 | 4.97 ± 2.31 | 0.581 |
| *Ruminococcaceae UCG-005* | 7.29 ± 1.43 | 5.02 ± 1.72 | 0.340 |
| *Roseburia* | 1.51 ± 0.96 | 0.85 ± 0.19 | 0.516 |
| *Muribaculaceae_norank* | 2.49 ± 0.68 | 8.34 ± 1.75 | 0.014 |
| *Streptococcus* | 1.43 ± 0.72 | 0.29 ± 0.12 | 0.190 |
| *Rikenellaceae RC9 gut group* | 1.62 ± 0.35 | 2.77 ± 0.78 | 0.228 |
| *Enterococcus* | 0.70 ± 0.51 | 0.10 ± 0.08 | 0.282 |
| *[Eubacterium] coprostanoligenes group* | 2.74 ± 0.82 | 1.89 ± 0.47 | 0.392 |
| *Phascolarctobacterium* | 1.24 ± 0.50 | 1.85 ± 0.51 | 0.416 |
| *Ruminococcaceae UCG-002* | 7.63 ± 2.13 | 1.10 ± 0.49 | 0.036 |
| *Christensenellaceae R-7 group* | 17.28 ± 5.74 | 1.84 ± 0.49 | 0.054 |
| *Alloprevotella* | 0.66 ± 0.31 | 7.56 ± 2.29 | 0.039 |
| *Clostridium sensu stricto 1* | 1.34 ± 0.15 | 1.06 ± 0.44 | 0.564 |
| *Blautia* | 1.42 ± 0.22 | 2.03 ± 0.50 | 0.298 |
| *Ruminococcaceae_uncultured* | 2.61 ± 0.32 | 1.39 ± 0.29 | 0.022 |
| *Lachnospiraceae_uncultured* | 0.95 ± 0.32 | 2.67 ± 0.89 | 0.107 |
| *Paludibacteraceae_uncultured* | 0.39 ± 0.25 | 0.09 ± 0.06 | 0.261 |
| *Family XIII AD3011 group* | 1.61 ± 0.36 | 0.60 ± 0.19 | 0.036 |
| *Agathobacter* | 0.56 ± 0.22 | 0.60 ± 0.32 | 0.920 |
| *Coprococcus 3* | 0.30 ± 0.07 | 1.12 ± 0.26 | 0.032 |
| *Prevotella 1* | 0.42 ± 0.16 | 2.79 ± 1.39 | 0.129 |
| *Erysipelotrichaceae_uncultured* | 0.42 ± 0.15 | 1.28 ± 0.50 | 0.136 |
| *Prevotellaceae NK3B31 group* | 0.75 ± 0.18 | 5.33 ± 2.41 | 0.130 |
| *Lachnospiraceae_Unclassified* | 0.25 ± 0.07 | 0.15 ± 0.02 | 0.213 |
| *Turicibacter* | 0.58 ± 0.10 | 0.53 ± 0.23 | 0.831 |
| *Ruminococcus 2* | 0.49 ± 0.12 | 0.80 ± 0.27 | 0.326 |
| *Desulfovibrio* | 1.03 ± 0.16 | 0.32 ± 0.13 | 0.009 |

^1^ NF, sows fed with a semi-purified basal diet (0.01% dietary fiber); F, sows fed with a semi-purified basal diet supplemented with 8.33 g/kg inulin and 200 g/kg cellulose.

Values are mean ± standard error (*n* = 5). Differences between treatments were considered significant at *P* < 0.05.
